# The Many Faces of SetDB1

**DOI:** 10.3390/epigenomes10020024

**Published:** 2026-04-01

**Authors:** Stanislav E. Romanov, Dmitry E. Koryakov

**Affiliations:** 1 Institute of Molecular and Cellular Biology, Novosibirsk 630090, Russia; romanov@mcb.nsc.ru; 2 Department of Natural Sciences, Novosibirsk State University, Novosibirsk 630090, Russia

**Keywords:** SetDB1, H3K9 methylation, protein methylation, DNA methylation, PcG proteins

## Abstract

The conserved protein SetDB1 has been identified in various vertebrate and invertebrate groups. It plays key roles in vital processes such as germline and nervous system development, immune response, tumorigenesis, cell cycle progression, and others. SetDB1 is initially characterized as an enzyme that methylates lysine 9 on histone H3, leading to gene silencing, which is traditionally considered its primary function. However, SetDB1 also targets about a dozen nuclear, cytoplasmic, and membrane proteins as substrates. Moreover, some functions of SetDB1 do not require methyltransferase activity. Due to its SUMO-interacting motif, Tudor domain, and methyl-binding domains, SetDB1 interacts with a wide range of complexes that regulate protein stability and activity, signal transduction pathways, and chromatin spatial organization. In this review, we aim to expand the classical view of SetDB1 as solely a histone methyltransferase and to highlight the broader diversity of its functions.

## 1. Introduction

**Figure 1:**
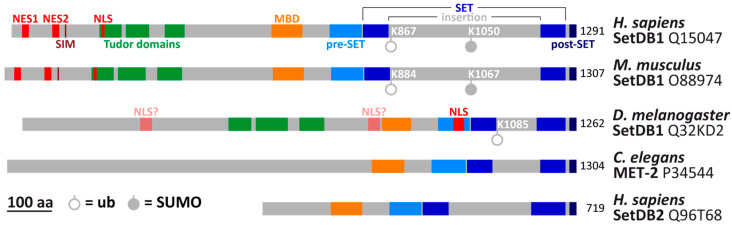
Domain organization of SetDB1 and SetDB2 molecules in different organisms. Protein lengths and domain positions are based on information from the UniProt database (Release 2024_04), with additions from the following articles: [10,24,27,30,31,32]. The UniProt sequence number is indicated after each protein name. Positions of ubiquitinated lysines (K867, K884, K1085) are taken from [30,33,34]. Note that in the original article on mouse SetDB1, the ubiquitination site is reported as K885; however, in the UniProt sequence O88974, this lysine is at position 884. SUMOylated lysines (K1050, K1067) are from [35] and the PhosphoSitePlus database [36]. NES—nuclear export signal; NLS—nuclear localization signal; SIM—SUMO-interacting motif; MBD—Methyl-CpG-Binding Domain; aa—amino acids; ub—ubiquitination site; SUMO—SUMOylation site.

SetDB1, also known as KMT1E (Lysine Methyltransferase 1E), is a conserved protein first identified in humans [1,2], followed by its discovery in mice (referred to as Eset) [3], the nematode *Caenorhabditis elegans* (known as MET-2) [4,5], and *Drosophila* (as dSetDB1 or Eggless/Egg) [6,7]. In mammals, its paralog SetDB2 has also been characterized; it differs from SetDB1 in size, domain composition, and expression profile across various tissues [8,9,10] (Figure 1).

The activity of SetDB1 is essential for cell proliferation, differentiation, fertility, and development. For example, SetDB1 mutations in *Drosophila* lead to female sterility and significantly reduce the viability of imagoes [7,11,12]. In *C. elegans*, the absence of MET-2 causes ectopic expression of germline genes in somatic cells [13]. In mice, a mutation in SetDB1 exhibits defective growth of the inner cell mass and the incapability of deriving stem cell lines in the embryo [14], whereas conditional deletion of SetDB1 severely impairs male and female meiosis [15,16,17], as well as development of various somatic tissues [18,19]. Aberrant expression of SetDB1 in humans is implicated in the pathogenesis of various diseases, predominantly cancer [20,21].

SetDB1 possesses a highly conserved SET domain, which is divided into two subdomains by a long insertion, giving the protein its name—SET Domain Bifurcated 1 [1] (Figure 1). The function of this domain is the methylation of lysine side chain, with the first identified substrate of SetDB1 being lysine 9 in histone H3 (H3K9) [2,3]. This epigenetic modification is most often regarded as SetDB1’s primary activity, and its absence is considered responsible for the disturbances observed in mutants. However, functions of SetDB1 extend beyond H3K9 methylation. The enzyme contains several domains (Figure 1) that significantly broaden its range of interactions with other proteins. Due to the presence of nuclear localization and nuclear export signals, SetDB1 can shuttle between the cytoplasm and nucleus [22,23,24,25,26,27], indicating that some of its functions occur outside chromatin. However, even when associated with chromosomes, SetDB1 does not always colocalize with H3K9 methylation [26,28,29], suggesting that it either modifies other proteins or performs functions independent of its enzymatic activity.

## 2. SetDB1 and H3K9 Methylation

SetDB1 methylates lysine 9 on free histone H3 in the cytoplasm and also modifies H3 within nucleosomes in the nucleus. In the cytoplasm, SetDB1 adds the first and partially the second methyl groups to H3K9 [22,23,25], a process that may occur during translation when the newly synthesized H3 molecule is still associated with the ribosome [37]. In the nucleus, SetDB1 adds the third methyl group to H3K9, a step that requires interaction with the conserved protein ATF7IP (MCAF1) in humans, or its orthologs mAM in mice, LIN-65 in nematodes, and Wde in *Drosophila* [27,30,38,39,40,41].

ATF7IP modulates SetDB1’s enzymatic activity, shifting it from mono-/dimethylation to trimethylation, and facilitates its binding to chromosomes [42,43]. Additionally, ATF7IP stimulates the ubiquitination of a conserved lysine within the SET domain insertion (K867 in humans, K884 in mice, and K1085 in *Drosophila*) [30,41] (Figure 1), which is essential for SetDB1’s catalytic activity [33,34,44]. Through the interaction of two ATF7IP molecules with the N-terminus of SetDB1, nuclear export signals are blocked [27,41,45], preventing SetDB1 from binding to the nuclear export receptor CRM1 (also known as XPO1) and thereby retaining SetDB1 in the nucleus [24,25,27,45]. In male germline cells, the ATF7IP ortholog ATF7IP2 (MCAF2) similarly forms a complex with SetDB1 [45,46,47].

In mammals, the targets of SetDB1 include both unique and repetitive sequences, among which endogenous retroviruses (ERVs) constitute a significant proportion. H3K9 methylation, primarily established by SetDB1, is the main mechanism for ERV inactivation in mouse embryonic stem cells (mESCs) [48,49,50]. In somatic cells, such as developing B lymphocytes, T lymphocytes, and brain cells, ERV silencing occurs through SetDB1-mediated H3K9 methylation in conjunction with DNA methylation [51,52,53,54].

**Figure 2:**
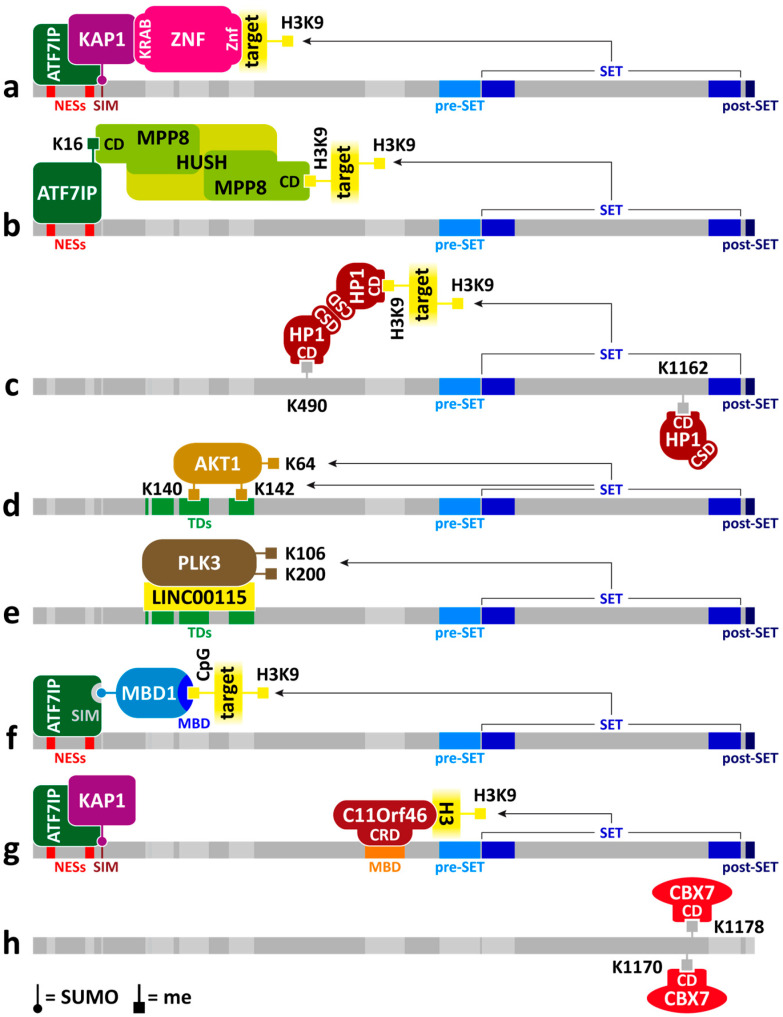
Interaction schemes of SetDB1 with other proteins, DNA, and RNA, using human SetDB1 as an example. Znf—zinc-finger domain; CD—chromodomain; CSD—chromoshadow domain. Arrows indicate lysines in proteins to which SetDB1, via its SET domain, attaches methyl groups. (**a**–**h**) represent variants of interactions between proteins. Other designations and detailed explanations are provided in the text.

DNA motifs within ERVs serve as recognition sites for a large family of proteins containing zinc-finger domains—ZNF or ZFP. Through their KRAB domain, ZNF/ZFP proteins bind the SUMO ligase KAP1 (also known as TRIM28), which recruits SetDB1 and other chromatin modifiers to ERVs, thereby inducing H3K9 methylation and ERV silencing [50,55,56]. The interaction between KAP1 and SetDB1 is mediated by the autoSUMOylated bromodomain in KAP1 and the SUMO-interacting motif at the N-terminus of SetDB1 [31] (Figure 1 and Figure 2a). In *Drosophila*, a similar process occurs, with SetDB1 binding to the SUMOylated ortholog of KAP1—Bonus [57].

Another mechanism for target inactivation involves the interaction of SetDB1/ATF7IP with the Human Silencing Hub (HUSH) complex [43,58,59]. One subunit of the HUSH complex, MPP8, contains a chromodomain that recognizes K9me3 in H3, K16me3 in ATF7IP, and K1170me3 in SetDB1 [60,61,62,63]. MPP8 partially colocalizes with SetDB1/ATF7IP at genes and various types of repeats, and deletion of either SetDB1 or MPP8 results in activation of these sequences [64].

There are different models describing the interaction between SetDB1 and the HUSH complex. For example, SetDB1 methylates H3K9, after which MPP8 binds to the methylated site via its chromodomain. This establishes an inactive chromatin state, and for its maintenance, only the HUSH complex is required, while H3K9me3 and SetDB1 are no longer necessary [65]. According to another model, MPP8, upon binding to H3K9me3, recruits SetDB1/ATF7IP to the locus to maintain and spread H3K9 methylation [58]. Different MPP8 molecules within the HUSH complex perform distinct functions, with one binding to H3K9me3 and another interacting with SetDB1/ATF7IP to methylate H3K9 on neighboring nucleosomes [63] (Figure 2b).

In addition to repetitive sequences, SetDB1 targets tissue- and stage-specific genes [28,51,66,67,68,69,70,71]. Several mechanisms recruit SetDB1 to these targets, which may be similar to those involved in repetitive sequence regulation, as described above [2,64,65,72]. Additionally, various transcription factors can mediate SetDB1 recruitment; for example, SUMOylated Sp3 recruits SetDB1 to promoters through interaction with its SUMO-interacting motif [73]. Another transcription factor, RelB, recruits two methyltransferases, SetDB1 and G9a, to the *interleukin 17* locus [74], while Smad3 recruits SetDB1 and Suv39h1 to *interleukin 2* [75]. The multidomain transcription factor POGZ recruits SetDB1/KAP1 to ERVs and the *Dux* gene promoter in mESCs [76], as well as to telomeres of ALT-positive (Alternative Lengthening of Telomeres) human tumor cells [77]. The binding of SetDB1 to both genes and ERVs can also be mediated by JARID1B or JARID2 [78,79]. Although these proteins belong to the family of demethylases, JARID2 is catalytically inactive due to its structural features [80], and the interaction of SetDB1 with JARID1B is independent of its demethylase activity [79]. The list of proteins recruiting SetDB1 to chromatin can be extended further.

An important prerequisite for H3K9 methylation is the SUMOylation of a lysine residue in the middle of the SET domain (K1050 in humans or K1067 in mice, Figure 1). Without this modification, H3K9 methylation does not occur [35,81]. SUMOylation does not affect SetDB1’s nuclear entry or catalytic activity but is necessary for its binding to chromatin [82]. Another study found that the same lysine, K1050 (along with K182), in human SetDB1 can be polyubiquitinated, leading to proteasomal degradation of the protein. To prevent this, the C-terminus of SetDB1 interacts with the protein IRTKS, which recruits the deubiquitinase OTUD4 to SetDB1, removing the polyubiquitin chain [83].

## 3. SetDB1 and Active Histone Marks

Examples from the previous section fit into a model in which SetDB1, through various intermediaries, interacts with chromatin and methylates H3K9, leading to target inactivation. In this context, the presence of H3K9me2/me3 usually implies the absence of modifications characteristic of active transcription. However, this representation is a significant simplification, and relationships of SetDB1 with different histone modifications are far from straightforward.

SetDB1 is not the only enzyme that methylates H3K9; for example, in *Drosophila*, at least two other such enzymes are known (Su(var)3–9 and G9a), and in humans, there are nine more (SUV39H1, SUV39H2, G9A, GLP, PRDM2, PRDM3, PRDM8, PRDM16, and SetDB2, which is already mentioned above) [84]. Analysis of SetDB1 profiles on the chromosomes of mice, humans, and *Drosophila* has revealed regions where SetDB1 is present but H3K9 methylation is absent [26,28,29,72]. In other words, SetDB1 is neither necessary nor sufficient by itself for the establishment of H3K9me2/me3 marks across the entire genome. Moreover, in regions lacking H3K9me3, SetDB1 can colocalize with active chromatin marks [26], and SetDB1 targets can be transcribed [29,66]. It is also worth noting that SetDB1-mediated H3K9 methylation can have different functions and, under certain conditions, may even promote active expression. For example, the silencing of transposable elements in the *Drosophila* female germline is ensured by a piwiRNA-based mechanism [85], a key step of which is the transcription of piwiRNA clusters that requires SetDB1-dependent H3K9 methylation [86]. Interestingly, H3K9me2 is also present in these clusters in somatic cells, but there the methylation depends on Su(var)3–9, and the clusters remain inactive [87].

In undifferentiated mammalian cells, the promoter regions of some genes encoding transcription factors contain both active and repressive marks simultaneously. Such bivalent domains maintain low-level transcription of genes while keeping them poised for rapid activation. The first identified bivalent domains contained H3K4 and H3K27 methylation [88], and later domains with H3K4me3 and SetDB1-mediated H3K9me3 were discovered [89]. Additionally, SetDB1 is found in domains where H3K9 methylation and H3K14 acetylation coexist [32,90].

Another type of domains combines H3K36 and SetDB1-mediated H3K9 methylations [91]. These domains may have different functions. For example, they were found in the 3′ regions of transcriptionally active human genes encoding ZNFs. In this case, H3K9me3 is not involved in repressing transcription but rather serves to create a chromatin structure that can inhibit inappropriate homologous recombination between different highly related ZNF genes [92]. The same pair of modifications is also required for alternative lengthening of telomeres [93]. Colocalization of H3K9me3 and NSD1/2/3-mediated H3K36me2/me3, called dual domains, occurs in some lowly expressed genes and specific enhancers in human cells and mESCs [91,94].

Both bivalent and dual domains are not simply overlaps of different modifications; there is a functional connection between them, forming a special type of chromatin. For example, H3K9me3 and H3K36me3 coexist on the same histone tail, and removal of SetDB1 leads to the loss of both modifications [91]. H3K14 acetylation in *Drosophila* and mammals is required for SetDB1 recruitment and subsequent H3K9 methylation on the same nucleosome [95,96]. Different types of bivalent domains may also be interconnected. For instance, in mESCs, the promoters of the adipogenic master regulatory genes *Cebpa* and *Pparg* are enriched with H3K27me3 and H3K4me3. In preadipocytes, H3K4me3 is retained upstream of transcription start sites, while H3K27 methylation is replaced by SetDB1-mediated H3K9 methylation downstream of transcription start sites [89].

## 4. SetDB1 and Methylation of Non-Histone Proteins

In addition to histone H3, SetDB1 has several other nuclear, cytoplasmic, and transmembrane proteins as substrates. By attaching methyl groups to these proteins, SetDB1 influences their activity or stability. Moreover, in humans, SetDB1 is capable of automethylation at lysine residues K490 and K1162. These modifications do not affect SetDB1’s catalytic activity but are necessary for its interaction with the HP1 protein, which is required for the subsequent maintenance and spreading of H3K9me3 [97] (Figure 2c).

SetDB1 modulates the activity of p53- and PI3K/AKT-dependent pathways, playing important roles in transducing pro-apoptotic and anti-apoptotic signals, respectively [98]. SetDB1 dimethylates lysine 370 in the transcription factor p53, thereby stimulating its phosphorylation at serine 15, which protects p53 from ubiquitination and degradation [99]. But besides that, SetDB1 can directly repress *Trp53* activity (the gene encoding p53) by methylating H3K9 at its promoter region, leading to transcriptional silencing [100].

One component of the PI3K/AKT-dependent pathway is the serine/threonine kinase AKT1, in which SetDB1 trimethylates lysines K140 and K142. These modifications promote subsequent phosphorylation of AKT1 at positions T308 and S473, enhancing its kinase activity. The methylated lysines K140 and K142 serve as binding sites for the Tudor domains of SetDB1, allowing it to methylate another site in AKT1—lysine K64 [101] (Figure 2d). This modification recruits the demethylase JMJD2A, which acts as a bridge between AKT1 and the ubiquitin ligases TRAF6 and Skp2-SCF. This sequence of events promotes AKT1 ubiquitination, recruitment to the cell membrane, and activation [102].

The next pathway involving SetDB1 includes the hypoxia-inducible transcription factor HIF-1α, which regulates numerous effector genes governing proliferation, energy metabolism, invasion, and metastasis [103]. The long non-coding RNA *LINC00115* acts as a scaffold, facilitating the binding of SetDB1 (via its Tudor domain) and the serine/threonine kinase PLK3. SetDB1 methylates lysines K106 and K200 in PLK3, suppressing its kinase activity and thereby preventing PLK3-mediated phosphorylation of HIF-1α. This process increases HIF-1α stability [104,105] (Figure 2e).

Another pathway influenced by SetDB1 involves the transmembrane protein CD147 (also known as Basigin). SetDB1 dimethylates lysine K71 in CD147, triggering a cascade that leads to phosphorylation of the transcription factor p38, activation of the *FosB* gene, and induction of apoptosis [106]. Closely associated with CD147 is the lactate transporter MCT1, which is trimethylated by SetDB1 at lysine K473. This modification prevents MCT1 degradation, enhances lactate export, and promotes tumor cell glycolysis [107].

SetDB1 also modifies the transcriptional coactivator PC4, which regulates oscillations in mRNA levels of genes involved in cell cycle progression. For example, during the early G2 phase, PC4 binds the mRNA of the gene encoding the outer kinetochore component CENPF. This interaction recruits SetDB1, which dimethylates lysine K35 in PC4. Subsequently, PC4 associates with the RNA helicase UPF1, leading to degradation of CENPF mRNA in the late G2 phase [108].

SetDB1 regulates the transcription of ribosomal DNA. A key element in this process is the Upstream Binding Factor (UBF), which maintains an open chromatin state in the ribosomal gene cluster [109]. SetDB1 inactivates UBF through trimethylation of lysines K232 and K254, leading to chromatin condensation and reduced transcription levels [110]. In addition to the aforementioned proteins, SetDB1 also methylates the inhibitor of growth ING2 [111] and the regulatory protein Tat from human immunodeficiency virus [112]. Furthermore, substrates of SetDB1 appear to include the cytoplasmic kinase ASK1 [113], subunits of the ubiquitin ligase complex TRIM71, which is involved in RNA processing [114], as well as Serpinh1, a regulator of pro-collagen folding and maturation [115].

**Figure 3:**
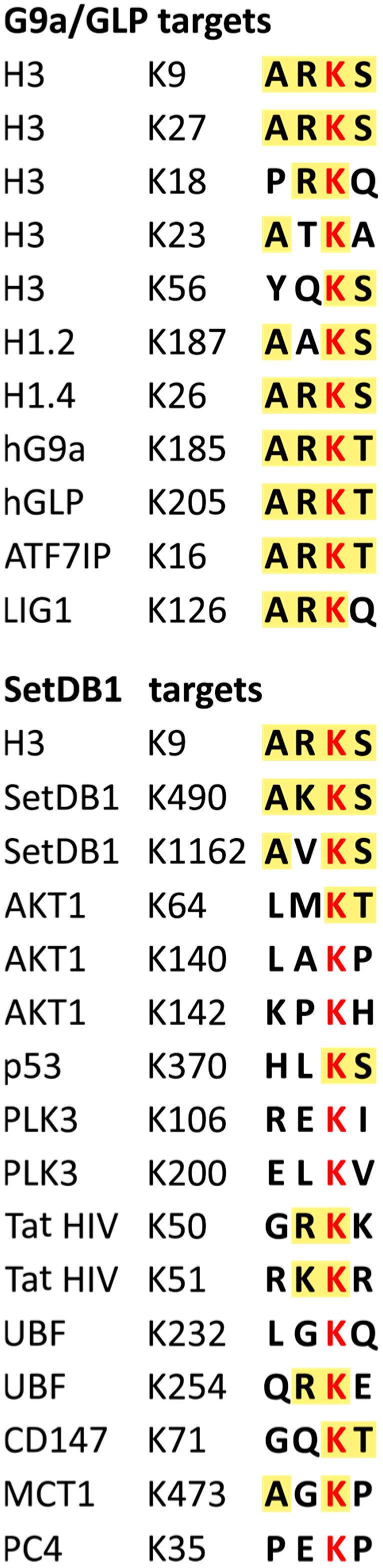
Motifs surrounding lysines methylated by G9a/GLP and SetDB1. The left column lists protein names, the middle column indicates the position of the modified lysine, and the right column shows the motif containing the modified lysine, emphasized in red. The background highlights amino acids matching the A(R/K)K(S/T) motif. Positions of methylated lysines and motif compositions are taken from [84,97,99,101,102,105,106,107,108,110,112,116], as well as from the UniProt database [117].

It is interesting to note that the activity of other H3K9-specific enzymes, such as G9a and GLP, is also not limited to H3K9. G9a and GLP tend to modify lysines that are part of the histone-mimic motif A(R/K)K(S/T), which resembles the H3K9-containing sequence. The amino acids surrounding SetDB1-methylated lysines match this motif only in SetDB1 itself, whereas in other proteins, the histone-mimic motif is degenerate or absent (Figure 3).

## 5. Methyl-CpG-Binding Domain of SetDB1

In the middle part of the SetDB1 molecule lies the Methyl-CpG-Binding Domain (MBD) (Figure 1), which suggests a potential direct link between H3K9 methylation and DNA methylation. Although this connection is often assumed, SetDB1 itself has not been observed to directly bind methylated DNA. Instead, this interaction is mediated by the protein MBD1, which recruits SetDB1 to DNA through its binding with ATF7IP [118,119,120] (Figure 2f).

SetDB1 directly interacts with the DNA methyltransferases DNMT3A and DNMT3B [121], and DNMT3L enhances the binding between DNMT3A and SetDB1 [122]. Loss of SetDB1 disrupts the telomeric binding of DNMT3A, DNMT3B, and DNMT3L [93]. Deletion of SetDB1 results in reduced H3K9 and DNA methylation levels at specific loci in mESCs [123]. However, genes upregulated in SetDB1-deficient mESCs, with few exceptions, do not overlap with those upregulated in cells deficient for DNMT1, DNMT3A, and DNMT3B [49]. Other studies show that DNMTs and SetDB1 act through distinct pathways to repress germ cell-related genes in mESCs [124], and triple knockout of DNMT1/3A/3B does not affect SetDB1 binding or SetDB1-dependent H3K9 methylation [48]. Thus, the interaction between DNA methylation and SetDB1-mediated H3K9 methylation cannot be explained by a simple model. Further complicating this relationship is the presence of the MBD in *Drosophila* SetDB1 and *C. elegans* MET-2, despite the long-held belief that DNA methylation is absent or extremely low in these organisms [125,126].

Insight into the function of the MBD is provided by a study investigating the 3D structure of this domain in human SetDB1 and SetDB2. The domain was found to be noncanonical, comprising a conserved core and a unique N-terminal extension. Consequently, the MBD has lost its ability to bind methylated DNA but has gained a protein–protein interaction surface that allows it to bind to C11orf46 (also known as ARL14EP) [127]. On the C11orf46 side, recognition of SetDB1 occurs through its cysteine-rich domain (CRD) [128]. It is highly likely that this interaction via the MBD and CRD also occurs in other organisms, as orthologs of C11orf46 are found in nematodes (ARLE-14) and *Drosophila* (CG14464). Moreover, interactions between these orthologs and MET-2 or SetDB1 have been detected [39,129,130].

It is hypothesized that C11orf46 acts as a mediator for the binding of the SetDB1/ATF7IP/KAP1 complex to chromatin, as proteins associated with C11orf46 include SetDB1, KAP1, ATF7IP, and histone H3. Furthermore, different regions of the C11orf46 molecule are responsible for interactions with SetDB1 and histone H3 [128] (Figure 2g). Similarly, in *C. elegans*, ARLE-14 helps recruit MET-2 to chromatin [39,40]. In *Drosophila*, the CG14464 protein has not yet been studied.

## 6. Tudor Domains of SetDB1

At the N-terminus of SetDB1 lie the Tudor domains (TDs) (Figure 1). Initial studies identified one or two TDs in SetDB1, but later, a tandem triple Tudor domain (TTD) was discovered. In addition to the proteins and RNAs previously mentioned, the TTD in mammalian SetDB1 can bind to histone H3 carrying both K14 acetylation and K9 methylation. TD3 preferentially recognizes K9me1 or K9me2, while TD2 selectively binds K9me3; TD1, however, is unable to bind methylated lysines. The region between TD2 and TD3 is responsible for interaction with K14ac [32]. The TTD in *Drosophila* SetDB1 has different properties, as it shares only 33% homology with the human SetDB1 TTD. The region recognizing H3K14ac retains the highest degree of homology, whereas TD2 and TD3 lack the amino acids required for interaction with H3K9me [96]. In mammals, binding of the TTD to H3K14ac or H3K9me separately is much weaker than binding to their combination [32], whereas in *Drosophila*, recognition of H3K14ac is independent of H3K9 methylation [96].

There is a functional link between the TTD and the SET domain. The synthetic molecule (R,R)-59 (also known as SETDB1-TTD-IN-1) binds to the TTD and completely prevents its association with H3 [131]. This interaction blocks methylation of lysines in H3 and ASK1 [113,132], but unexpectedly increases K64 methylation and subsequent T308 phosphorylation in AKT1 [133]. Another synthetic molecule, UNC10013, binds to the same TTD region as (R,R)-59 but inhibits the methyltransferase activity of SetDB1 toward AKT1 [134]. Two natural compounds—emetine from the plant *Carapichea ipecacuanha* and echinomycin from the actinomycete *Streptomyces echinatus*—also bind to the TTD and prevent H3K9 methylation [135,136].

The region of human SetDB1 between residues 279 and 329, where TD2 is located, can bind the chain of ubiquitin-like proteins Nedd8. This interaction enables SetDB1 to form a complex with the neddylated kinase PDK1 and other proteins, facilitating subsequent methylation and phosphorylation of AKT1 [137].

## 7. SetDB1 and Polycomb Group Proteins

In addition to H3K9-mediated silencing, SetDB1 is also associated with another epigenetic system involving H3K27 methylation and Polycomb group (PcG) proteins, which form several Polycomb Repressive Complexes (PRCs). In *Drosophila*, H3K27 methylation is carried out by the PRC2 complex, which contains the enzyme E(z) [138,139], and H3K27me3 serves as a docking site for the Polycomb (Pc) chromodomain within PRC1 [140,141]. In mammals, this system is more intricate because PcG proteins have multiple paralogs and form various complex variants. For example, there are six canonical and several non-canonical variants of PRC1 [142,143], and five types of Pc proteins: CBX2, CBX4, CBX6, CBX7, and CBX8 [144,145].

The interaction between SetDB1 and PcG proteins, as well as with H3K27me3, occurs through distinct mechanisms. For instance, their cooperative function may be seen as a “summation” of their repressive effects. A subset of developmental genes in mESCs is repressed by both PcG and SetDB1, with target genes enriched for both H3K9me3 and H3K27me3. Disruption of either PcG- or SetDB1-mediated repression can destabilize the stem cell state [66]. Additionally, a stepwise mechanism exists by which SetDB1 and PRC1.6 repress germline genes in mouse embryos, with PRC1.6 required for SetDB1-mediated H3K9 methylation at bound genes [146,147]. MGA, a scaffolding component of PRC1.6, recruits SetDB1 via its interaction with ATF7IP to meiosis-related genes in mESCs. Thus, MGA plays a dual role: it is central in establishing a PcG-dependent repressive state and subsequently strengthens repression by recruiting the SetDB1/ATF7IP complex to induce H3K9me3 modifications [148].

The relationship between SetDB1 and PcG may be more complex when one system indirectly regulates the activity of the other. For example, high expression of EZH2 (the mammalian ortholog of *Drosophila* E(z)) in cancer cells leads to H3K27 methylation at the *miR-381* promoter, suppressing its expression. Normally, *miR-381* represses SetDB1, but in the presence of excess EZH2, SetDB1 is aberrantly upregulated. Subsequently, SetDB1 methylates AKT1, thereby promoting cell proliferation [149,150,151]. Additionally, EZH2 suppresses the transcription factor RUNX3, which allows *SetDB1* expression. Inactivation of EZH2 upregulates RUNX3 and, consequently, downregulates SetDB1 [152]. SetDB1 also regulates EZH2: SetDB1 knockdown reduces EZH2 expression, whereas SetDB1 overexpression increases EZH2 levels. Furthermore, SetDB1, in complex with the deubiquitinase USP11, protects several proteins—including EZH2—from ubiquitination and degradation. Notably, this protective function is attributed to the N-terminus of SetDB1 and does not require its catalytic SET domain [153].

Another mode of interaction between the two systems involves the non-canonical activities of their components. As mentioned earlier, SetDB1 binding sites on mESC chromosomes are heterogeneous, with some regions containing H3K9 methylation and others lacking it. In regions without H3K9me3, SetDB1 may associate with PRC2 and H3K27 methylation, and SetDB1 removal impairs EZH2 binding and reduces H3K27me3 levels [28].

On human and mouse chromosomes, sites containing CBX7/Cbx7 have been identified in the absence of both H3K27me3 and H3K9me3 [69,145,154]. This suggests an alternative mechanism for CBX7/Cbx7 recruitment that does not require histone methylation. SetDB1 may participate in this process, as it has been found among CBX7 binding partners [69]. Moreover, interaction with SetDB1 requires a functional chromodomain of CBX7 [136], and two methylated lysines in SetDB1 (K1170 and K1178) bind CBX7 in vitro with even greater affinity than H3K27me3 [155,156] (Figure 2h).

## 8. Noncatalytic Functions of SetDB1

The number of studies identifying functions of SetDB1 that do not require its methyltransferase activity is gradually increasing. For example, MET-2 in *C. elegans* can inactivate its targets through both catalytic and non-catalytic mechanisms [157]. In mice, three SetDB1 transcripts with different lengths and expression profiles have been characterized. The shortest transcript produces a protein lacking the MBD, pre-SET, SET, and post-SET domains [158]. The full-length SetDB1 forms a complex with deacetylases HDAC1 or HDAC2 and transcription corepressors Sin3A or Sin3B, which inhibits the activity of a reporter gene. The short isoform of SetDB1 contains only Tudor domains but is still capable of forming such a complex, retaining its inactivating ability, which in this case depends on HDAC1/2 and Sin3A/3B. This property is also not lost in SetDB1 mutants with a key amino acid substitution in the SET domain or lacking this domain entirely [159].

For the enzymatic function of SetDB1, ubiquitination of a conserved lysine in the SET domain (K884 in mice, Figure 1) is necessary; substitution of this lysine with alanine abolishes SetDB1’s methyltransferase activity. In preadipocytes, this mutant protein binds its targets, but some genes (e.g., *Tril* and *Gas6*) lose H3K9me3 and become activated, while others (e.g., *Cebpa* and *Pparg*) retain H3K9me3 and remain inactive. In the latter case, methylation is carried out by Suv39h1/h2, which bind these genes together with SetDB1 [34]. Given that the complete removal of SetDB1 leads to activation of *Cebpa* and *Pparg* [89], the presence of SetDB1 itself, even if catalytically inactive, appears important for forming the methylating complex with Suv39h1/2. The opposite situation can be observed in human centromeres, where SetDB1 and SUV39H1/2 are primarily responsible for H3K9 methylation. However, if these enzymes are removed, methylation is performed by the G9a/GLP complex. Notably, the presence of even catalytically inactive SetDB1 blocks the binding of G9a/GLP to centromeres and prevents H3K9 methylation there. This reveals a catalytic-independent role of SetDB1 in safeguarding centromere identity by restricting G9a/GLP-mediated methylation and preventing aberrant chromatin expansion [160].

SetDB1 modulates the degradation of the key transcriptional regulator Rb. Through KAP1 activity, Rb is ubiquitinated at lysine K810 and subsequently degraded. This same residue can be methylated; SetDB1 binds to K810me via its Tudor domains, preventing Rb ubiquitination and degradation. In this case, SetDB1 does not methylate Rb but merely interacts with K810me independently of the SET domain [161].

The cytoplasmic fraction of SetDB1 exhibits non-catalytic functions as well. In *Drosophila*, SetDB1 colocalizes with spindle microtubules (MTs). Both deletion and overexpression of SetDB1 affect MT dynamics, with overexpression of either the wild-type or catalytically inactive mutant equally altering MT polymerization rates [162]. α-Tubulin, which forms MTs, can be modified by acetylation or deacetylation at lysine K40, the latter catalyzed by the deacetylase HDAC6 [163]. Although the biological role of this modification is not fully understood, it may influence MT stability and polymerization [164]. SetDB1 interacts with HDAC6, and deletion of SetDB1 increases tubulin acetylation levels in MTs. Thus, SetDB1’s influence on MT dynamics appears to be mediated by HDAC6 rather than its enzymatic activity [162].

In mammalian mitosis, the SetDB1 paralog SetDB2 is involved in chromosome segregation [9]. SetDB2 mutants exhibit increased abnormal spindle and chromosome segregation phenotypes, which are rescued by expression of either wild-type SetDB2 or a catalytically inactive mutant [165]. In the mammalian liver, SetDB2, together with the glucocorticoid receptor, activates a subset of genes in response to fasting, facilitating long-range enhancer–promoter interactions independently of its enzymatic activity [166].

## 9. SetDB1 and Nuclear Architecture

The interphase nucleus represents a hierarchical system of domains, ranging from large chromosomal territories to loops connecting the promoter of an individual gene with its enhancer. At the sub-megabase level, this hierarchy includes Topologically Associating Domains (TADs)—extended regions of self-interacting chromatin that are spatially insulated from neighboring TADs by well-defined boundaries. In the nuclei of mouse and human neurons, the configuration of some individual TADs depends on SetDB1, and its deletion or overexpression alters TAD configuration [167,168].

Multisubunit complexes containing Cohesin and insulator proteins play a key role in organizing both individual loops and entire TADs [169,170]. Experiments in mammals have shown that SetDB1 can interact with these complexes and, consequently, regulate the spatial organization of chromatin. In mESC chromosomes, regions were identified where the SetDB1/ATF7IP complex colocalizes with Cohesin. These regions are characterized by the presence of active and the absence of repressive epigenetic marks, including H3K9me3. Moreover, the binding of Cohesin and SetDB1/ATF7IP to these sites is interdependent and does not require the methyltransferase activity of SetDB1. Deletion of SetDB1 or ATF7IP alters chromatin topology in these regions and the expression of nearby genes [171].

SetDB1-mediated H3K9me3 competes with the insulator protein CTCF for binding to a subset of SINE elements in mouse chromosomes. In the absence of SetDB1 and H3K9me3 in these regions, CTCF occupancy significantly increases. Although higher-order chromatin structures, including TADs, remain intact, chromatin loops and local interactions are disrupted, leading to transcriptional changes [172,173,174]. In human lung carcinoma cells, loss-of-function mutation of SetDB1 does not alter the boundaries of most TADs. However, in specific loci where H3K9 methylation disappears, CTCF enrichment occurs, and large TADs split into smaller ones, altering the activity of genes within them [175].

In *Drosophila*, interphase chromosomes contain inactive TADs, separated by short boundaries or longer transcriptionally active inter-TADs. Each of these structures is associated with a specific set of histone modifications, such as H3K27me3 found in TADs [176]. Comparison of the SetDB1 profile in salivary gland chromosomes with the distribution of domains enriched in H3K27me3 revealed that SetDB1 is often located precisely at the boundaries of these domains [29]. However, the extent to which these boundaries depend on SetDB1 remains an open question.

In mammals, SetDB1 is a component of PML nuclear bodies (PML-NBs), which are involved in various intranuclear processes, although their function is not fully understood. PML-NBs may be regions of the nucleus where protein modification or storage/sequestration occurs, or they may regulate the binding of proteins to specific chromatin regions [177]. SetDB1 is a key component required for the formation of these bodies, alongside the protein PML (also known as TRIM19) [24,178]. Moreover, for the binding of SetDB1 to PML-NBs, a functional SUMO-interacting motif is necessary, but catalytic activity may not be required, as a variant lacking part of the pre-SET domain has been identified in PML-NBs alongside full-length SetDB1 [24].

## 10. Conclusions

SetDB1 has been identified in various model organisms and has been the subject of hundreds of studies, only a small fraction of which are mentioned in this review. Interest in SetDB1 is driven by its involvement in numerous vital processes and, in humans, by a strong association between SetDB1 dysregulation and poor tumor prognosis [20,21]. Therefore, the search for ways to control the activity of the *SetDB1* gene or SetDB1 protein represents a highly promising direction in anti-tumor therapy. This, in turn, necessitates the identification of factors that regulate *SetDB1* gene transcription, protein activity, as well as its partners and targets.

We have demonstrated that the functions of SetDB1 are diverse and not always directly linked to its methyltransferase activity. Therefore, attributing the effects of SetDB1 mutation or knockdown solely to changes in histone methylation patterns and transcription of its direct targets would be an oversimplification. When interpreting experimental results, it is important to keep in mind the potential influence of SetDB1 on other proteins and cellular systems. Furthermore, it is essential to consider the various mechanisms through which SetDB1 can affect specific processes, as illustrated by p53, where SetDB1 regulates both gene transcription and protein stability.

An area that has been scarcely covered in this review but is no less intriguing is the modification of SetDB1 itself. To date, only a handful of these modifications have been characterized in detail. However, according to the PhosphoSitePlus database [36], dozens of SetDB1 modifications have been identified, most of which lack information regarding the enzymes responsible for their establishment, the proteins that recognize them, and their influence on SetDB1’s activity or stability.

## Data Availability

Data sharing is not applicable. No new data were created or analysed in this study.
